# Perioperative glycemic control with a computerized algorithm versus conventional glycemic control

**DOI:** 10.1186/cc10786

**Published:** 2012-03-20

**Authors:** M Punke, S Bruhn, M Goepfert, S Kluge, H Reichenspurner, A Goetz, D Reuter

**Affiliations:** 1 University Medical Center Hamburg-Eppendorf, Hamburg, Germany; 2 University Heart Center, Hamburg, Germany

## Introduction

In critically ill patients, both hypoglycemia and hyperglycemia seem to influence outcome. Since hypoglycemia can lead to organ dysfunction, hyperglycemia seems to boost surgical site infections (SSI) [1]. It was shown that intensive insulin therapy (IIT) reduced mortality in critically ill patients [2]. Unfortunately several studies could not reproduce the effects [3,4]. In particular, IIT bears the risk of accidental hypoglycemia which could even have a negative effect on patient outcome [3,4]. In cardiac surgery, the use of blood cardioplegia for cardiopulmonary bypass frequently leads to high blood glucose levels during surgery. In particular, a computer-based algorithm that guides the insulin therapy might be beneficial. We hypothesized that in patients undergoing major cardiac surgery with cardiopulmonary bypass and blood cardioplegia, the use of a computer-based algorithm for the application of insulin will lead to a tighter adherence of normoglycemia. Our primary study end-point was the duration, in which the patients fulfilled the predefined target range of 80 to 150 mg/dl blood glucose. Patients with conventional blood glucose therapy served as controls.

## Methods

Seventy-five patients were enrolled and randomized into three groups. Start of therapy was determined as the beginning of cardiopulmonary bypass. Group 1: therapy with computer-based blood glucose control (TGC System; Braun, Melsungen, Germany) and measurement of blood glucose every 30 minutes. Group 2: same therapy as group 1 and measurement of blood glucose every 15 minutes. Group 3: conventional therapy using a fixed insulin dosing scheme. End of therapy was defined as discharge from the ICU. Statistical analysis was performed with using ANOVA and the LPS *post hoc *test. Data shown are mean ± standard deviation, *n *= number of patients.

## Results

There were no statistical differences between the groups regarding age, height, weight, premedical history or intraoperative amount of glucose administration during cardioplegia (33 ± 15 g). Blood glucose levels in groups 1 and 2 stayed significantly longer in the target interval compared with group 3 (75 ± 19% vs. 72 ± 19%; vs. 50 ± 34%, *P *< 0.01, *n *= 25, respectively). There was no significant difference between the groups regarding ICU or hospital stay and SSI rates.

## Conclusion

Early computer-based insulin therapy allows one to better warrant normoglycemia in patients undergoing major cardiac surgery with the use of blood cardioplegia.

## References

[B1] Ann Intern Med 2007 146 233 243 17310047 10.7326/0003-4819-146-4-200702200-00002

[B2] N Engl J Med 2001 345 1359 1367 10.1056/NEJMoa011300 11794168

[B3] N Engl J Med 2006 354 449 461 10.1056/NEJMoa052521 16452557

[B4] N Engl J Med 2009 360 1283 1297 19318384 10.1056/NEJMoa0810625

